# HA-tag CD63 is a novel conditional transgenic approach to track extracellular vesicle interactions with sperm and their transfer at conception

**DOI:** 10.1038/s41598-023-27898-5

**Published:** 2023-01-13

**Authors:** Christopher P. Morgan, Victoria E. Meadows, Ruth Marx-Rattner, Yasmine M. Cisse, Tracy L. Bale

**Affiliations:** 1 Department of Pharmacology and Center for Epigenetic Research in Child Health and Brain Development, University of Maryland School of Medicine, Baltimore, MD 21201 USA; 2 Department of Psychiatry, University of Colorado School of Medicine, CU Anschutz Medical Campus, 12800 E. 19th Avenue, Aurora, CO 80045 USA; 3 Present Address: The Anschutz Foundation Endowed Chair in Women’s Integrated Mental and Physical Health Research at the Ludeman Center, Aurora, USA

**Keywords:** Embryogenesis, Germline development, Reproductive biology

## Abstract

Extracellular vesicles (EVs) are a unique mode of intercellular communication capable of specificity in transmitting signals and cargo to coordinate local and distant cellular functions. A key example of this is the essential role that EVs secreted by epithelial cells lining the lumen of the male reproductive tract play in post-spermatogenic sperm maturation. We recently showed in a preclinical mouse model that this fundamental process had a causal role in somatic-to-germline transmission of biological information regarding prior stress experience capable of altering the rate of fetal development. However, critical mechanistic questions remain unanswered as to the processes by which signaling occurs between EVs and sperm, and whether EVs or their cargo are delivered at conception and are detectable in the early embryo. Unfortunately, notable methodological limitations shared across EV biology, particularly in the isolation and labeling of EVs, complicate efforts to answer these important questions as well as questions on EV targeting specificity and mechanisms. In our current studies, we developed a novel approach to track EVs using a conditional transgenic construct designed to label EVs via conditional Cre-induced hemagglutinin (HA) tagging of the EV endogenous tetraspanin, CD63. In our exhaustive validation steps, this internal small molecular weight tag did not affect EV secretion or functionality, a common problem found in the previous design of EV tags using larger molecular weight proteins, including fluorescent proteins. Utilizing a stably transfected immortalized epididymal epithelial cell line, we first validated key parameters of the conditional HA-tagged protein packaged into secreted EVs. Importantly, we systematically confirmed that expression of the CD63-HA had no impact on the production, size distribution, or surface charge of secreted EVs, nor did it alter the tetraspanin or miRNA composition of these EVs. We also utilized the CD63-HA EVs to verify physical interactions with sperm. Finally, using in vitro fertilization we produced some of the first images confirming sperm delivered EV cargo at conception and still detectable in the early-stage embryo. As such, this construct serves as a methodological advance and as a valuable tool, with applications in the study of EV function across biomedical research areas.

## Introduction

Extracellular vesicles (EVs) are small membrane-bound nanoparticles produced by most eukaryotic cells. EVs serve an important role in intercellular communication by delivering complex bioactive cargo, including proteins, lipids, and nucleic acids, to recipient cells with a high degree of specificity^1–3^. EVs serve to dynamically coordinate and synchronize cellular and tissue signaling and are a unique means to shape cellular development and function. One example from the field of reproductive biology is the essential role of EVs secreted by epididymal epithelial cells as an integral component of the male reproductive tract in the post-testicular maturation of sperm^4–8^. Sperm exiting the seminiferous tubules of the testes are functionally immature and require interaction with epididymal EVs to undertake their basic physiological function of traversing the female reproductive tract and fertilizing an oocyte. These sperm-EV interactions actively shape the protein, lipid, and sncRNA content associated with sperm^7,9–16^. In particular, sperm-associated sncRNA profiles display considerable plasticity, being substantially remodeled as the cells transit the epididymis^7,11–14,17^. These changes in the sperm sncRNA are required not only for the gain of functions typifying mature sperm (i.e., motility and fertilization potential), but also for normal embryonic development^18–20^.

Recent studies from our lab and others suggest that there is a co-opting of this cellular communication mechanism as an efficient strategy to transfer relevant information regarding the paternal preconception environment to alter the course, especially the rate, of embryo development at and following fertilization^11,21–29^. Environmental factors including diet, drugs of abuse, environmental toxicants, and stress or trauma experience can alter the sncRNA content of epididymal EVs^13,26–28,30,31^. We recently demonstrated a role for the somatic-to-germline transmission of information regarding paternal stress experienced prior to conception via epididymal EVs that are capable of impacting fetal development in a preclinical mouse model^27^. In this model, paternal preconception stress produced lasting histone and transcriptomic alterations in mouse epididymal epithelial cells in vivo, with corresponding persistent changes in the miRNA and protein composition of EVs secreted by immortalized DC2 mouse epididymal epithelial cells treated with the stress hormone, corticosterone, in vitro ^27^. Demonstrating causality, we also showed that sperm incubated with EVs collected from these corticosterone-treated DC2 cells produced offspring with the same phenotypic changes in neurodevelopment and adult stress reactivity observed in the offspring of preconception-stressed male mice^27^. Others have demonstrated similar effects where sperm incubation with isolated epididymal EVs from mice following chronic ethanol exposure altered offspring phenotypes, including anxiety-like and ethanol-related behaviors^26^.

Key questions remain as to the fundamental biological processes underlying this epididymal EV-dependent intergenerational communication, including the molecular cargo encoding paternal experience, how these cargo are specifically enriched in new EVs, the specific cell or tissue targets of these EVs, and how EVs are delivered to these targets^8,32,33^. However, technical limitations shared across EV biology, especially in the isolation and labeling of EVs, complicate efforts to answer these questions^33,34^. EV populations within the epididymal lumen in vivo are highly heterogeneous, differing in terms of their origin, at the level of cell type and, at a finer scale, biosynthetic pathway (e.g., exosomes generated within multivesicular bodies vs microvesicles that bud directly from the cell membrane)^8^. These differences are likely to translate into functional consequences expressed in observable variation in bioactive cargos and cellular targeting of secreted EVs. However, currently available isolation and labeling methods limit the field’s capacity to resolve these populations^2^.

Current strategies to label EVs are limited and often problematic in terms of their significant effects on the EV loading and secretion mechanisms. Attempts to visualize EV behavior/function in vivo generally involved labeling of EVs, either through exogenous labeling after isolation or endogenously through the application of transgenic strategies to express tagged EV surface proteins. Each approach has its own limitations^35,36^. Lipophilic fluorescent dyes or lipid-anchored fluorophores are the most common reagents used to label isolated EV preparations. However, these reagents are non-specific and often label all EV subtypes present, as well as common lipoprotein contaminants. This labeling is non-covalent, so there is a high risk of shuttling to other cell membranes that do not reflect EV biodistribution. Finally, these amphiphilic reagents can self-assemble into EV-like particles. Endogenous labeling of EVs with EV-enriched tetraspanin fusion proteins avoids many of these pitfalls, though common approaches come with their own drawbacks^35,36^. Tetraspanins (e.g., CD9, CD81, CD63) have been labeled with a fluorescent protein or luciferase of approximately the same size as the proteins targeted for labeling, and the expression of the tagged protein is generally driven by an exogenous promoter resulting in elevated levels and off-target expression^37–45^. Unfortunately, these manipulations have largely impacted the functionality of the tagged tetraspanins that play a fundamental role in EV biology and facilitate EV budding by inducing membrane curvature and regulating EV cargo by recruiting other functional proteins into vesicles^46,47^.

Therefore, as a first step toward examining epididymal EVs in the somatic-to-germline transmission of paternal stress experience in vivo, we developed a transgenic construct that would allow for cell-type specific labeling of EVs via Cre-induced hemagglutinin (HA) tagging of the endogenous CD63 protein. While genetic strategies have been used to label EVs in cell lines and rodent models, our construct has advantages over previously reported transgenes^37–45^. First, CD63 is a tetraspanin protein enriched in predominantly small MVB-derived EVs (i.e., exosomes) widely expressed in tissues throughout the body, and we have designed our construct to be introduced via homologous recombination^40,46,48–52^. This knock-in approach avoids changes to the genomic context of CD63, retaining its endogenous regulation to avoid significant changes in EV production observed with other transgenes driven by synthetic promoters^37–39,42^. In addition, the HA tag (~ 3.3 kD) is an order of magnitude smaller than a fluorescent protein and we hypothesized would be less likely to impact the normal function of CD63. As such, this transgene could serve as a valuable tool with applications in the study of EV function across biomedical fields. We validated this construct in a stably transduced immortalized mouse caput epididymal epithelial DC2 cell line, confirming its ability to respond appropriately to Cre recombinase and express an HA-tagged CD63 that is trafficked to EVs for secretion. In addition, we examined HA-tagged CD63 EVs for changes in key biophysical characteristics (quantity, size distribution, and surface charge) and molecular composition (tetraspanin and miRNA content). Finally, we utilized these HA-tagged CD63 positive EVs to ask a biological question regarding physical interaction and passage of cargo following in vitro fertilization.

## Results and discussion

### Stably transfected DC2 epididymal epithelial cell line produces CD63-HA tagged EVs

**Figure 1:**
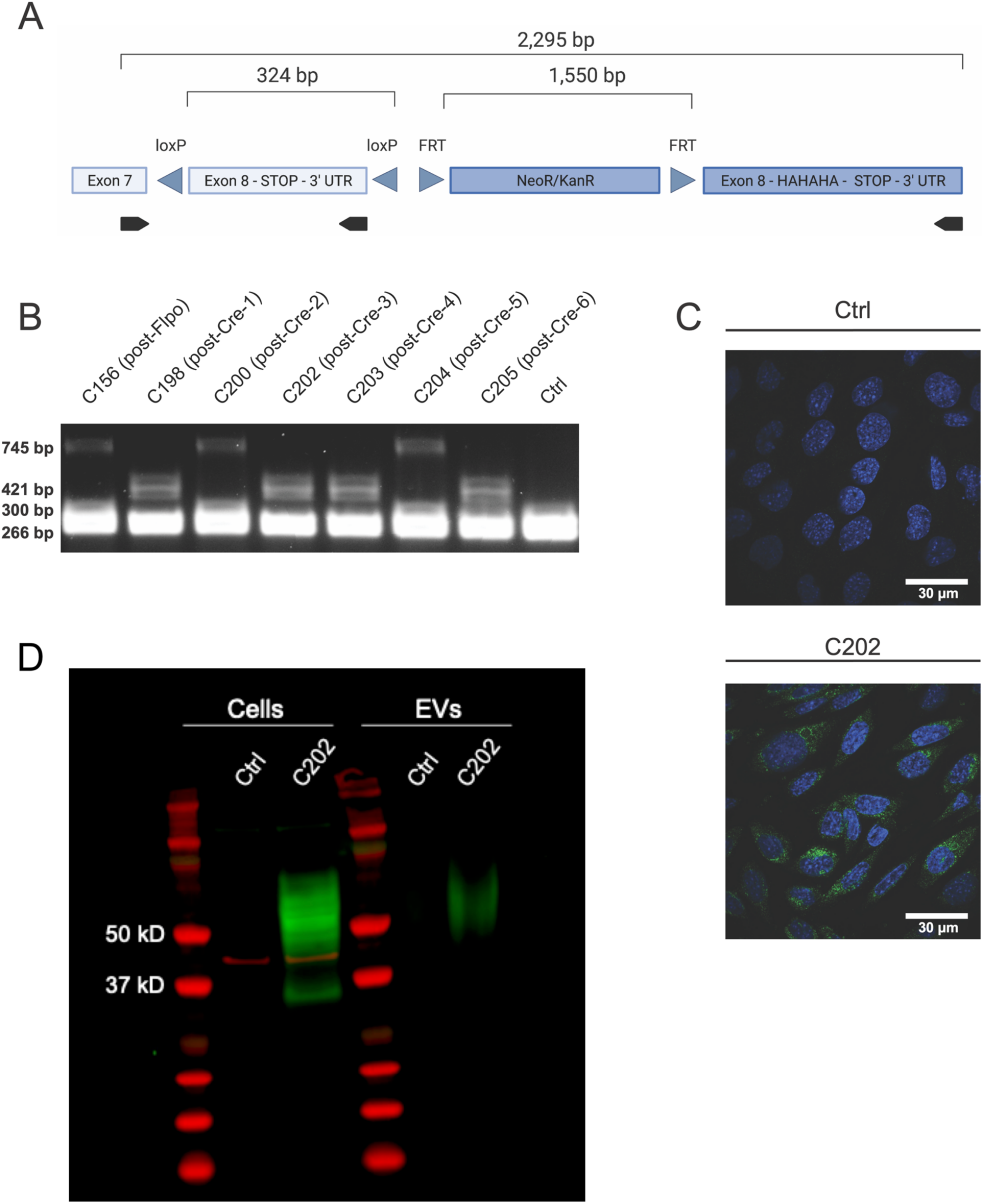
The CD63-HA transgene responds appropriately to Cre-recombinase expression, resulting in HA labeling of transfected DC2 epididymal epithelial cells and secreted extracellular vesicles (EVs). (**A**) Diagram of CD63-HA transgene. Following homologous recombination and removal of the NeoR/KanR cassette with flipase (Flpo), this transgene is designed to produce a fusion protein consisting of the EV-enriched tetraspanin CD63 with a carboxy terminal 3xHA-tag, under control of its endogenous promoter, in response to Cre recombinase. Endogenous and exogenous sequences are indicated by light and dark blue shading, respectively. PCR primer binding sites are indicated by arrows (black) below the diagram and the sizes of the largest possible amplicon and fragments expected to be removed upon appropriate Flpo and Cre-recombination are listed. (**B**) PCR amplification of CD63 from stably transfected DC2 epididymal epithelial cells shows the transgene recombined appropriately in response to Cre expression in 4 of 6 clonal cell lines derived from the parental C156 (post-Flpo) cell. Sanger sequencing confirmed the fidelity of this recombination and the lack of any mutations in C202, which was used for all subsequent experiments. A 266 bp amplicon corresponding to the wildtype CD63 allele is present in all samples, including control non-transfected DC2 cells (Ctrl). (**C**) ICC detects anti-HA staining specifically in the C202, but not in Ctrl cells. The pattern of anti-HA staining (green) is characteristic of CD63, suggesting the HA-tag does not affect the subcellular distribution of CD63-HA. Nuclei were counterstained with Hoechst 33342 (blue). (**D**) Western blot of whole cell lysates (cells) and EVs. Detection of a ~ 50–75 kD HA-tagged protein (green) was specific to C202 cells. The size range is consistent with the variably glycosylated CD63 protein. Actb protein (red) was used as a loading control. Similarly, an HA-tagged protein (green) of ~ 50–75 kD by western blot consistent with CD63 in isolated EVs from conditioned media from C202, but not Ctrl, cells demonstrate CD63-HA is successfully packaged into EVs for secretion. Total protein was extracted from a constant number of EVs (2.1 × 10^10^) from both cell lines, as determined by nanoparticle tracking.

CD63 is a tetraspanin enriched in EVs, especially exosomes, and is therefore frequently used as an EV marker protein. An inducible tagged CD63 would facilitate identifying EV sources and targets in vitro and in vivo. Therefore, we developed a targeting plasmid containing a transgene that would produce a carboxy terminal HA-tagged CD63 under control of its endogenous promoter in response to Cre-recombinase (Cre), based on the design of the Rpl22HA ‘RiboTag’ transgene (Fig. 1A)^53^. Our transgene includes an endogenous copy of the terminal CD63 Exon 8, which encodes a STOP codon and 3′ UTR, flanked by LoxP sites. This endogenous Exon 8 is followed by a second copy of Exon 8 encoding the addition of 3 consecutive HA tags at its terminus. Upon Cre-dependent recombination, the endogenous Exon 8 is removed and substituted with the HA-tag encoding exogenous Exon 8. The plasmid also contains flanking sequences for targeting to the CD63 locus via homologous recombination and a neomycin/kanamycin resistance (NeoR/KanR) cassette for G418 selection.

To facilitate mechanistic studies of epididymal EV-dependent intergenerational communication, we stably transfected a previously immortalized epididymal epithelial ‘DC2’ cell line with our CD63-HA transgene^54^. Following 8 weeks of antibiotic selection, the NeoR/KanR cassette was removed with flipase (Flpo), then a clonal post-Flpo CD63-HA cell line (C156) was isolated and transfected with Cre. To confirm appropriate recombination, post-Cre clonal cell lines were screened by PCR (Fig. 1B). Anticipated amplicons from post-Flpo cell lines include a 745 bp product from the CD63-HA transgene containing both the floxed endogenous and exogenous copies of Exon 8, a 300 bp product containing only the floxed endogenous Exon 8, and a 266 bp amplicon corresponding to the wildtype CD63 allele. Anticipated amplicons from transfected cell lines following Cre-recombination include a 421 bp product from the CD63-HA transgene containing the HA-tag encoding exogenous Exon 8 and a 266 bp amplicon corresponding to the wildtype CD63 allele. PCR amplification of CD63 shows the transgene recombined appropriately in 4 of 6 clonal cell lines derived from the parental C156 cell line following Cre expression. Sanger sequencing confirmed the fidelity of this recombination and the lack of any mutations in C202, which was used for all subsequent experiments^58^.

The expression and intracellular localization of CD63-HA in C202 cells was assessed by immunocytochemistry (ICC) (Fig. 1C). Anti-HA staining was present specifically in C202 cells and not control DC2 cells (Ctrl). In addition, the perinuclear localization of anti-HA staining is consistent with the expected distribution of CD63 in late endosomes and MVBs, suggesting the HA-tag does not impact the subcellular distribution of CD63^40,50,51^. To ensure the HA staining visualized via ICC was associated specifically with intact CD63, whole cell lysates and secreted EVs were analyzed by western blot (Fig. 1D, Supplemental Fig. 1A). Western blot detected an HA-tagged protein (~ 50–75 kD) in C202 cell lysate, which was absent in Ctrl cells. This size range is consistent with previous reports of the highly and variably glycosylated CD63 protein^49,55–57^. Finally, to verify HA-tagged CD63 is successfully trafficked to EVs and secreted, we performed a western blot on protein extracted from EVs isolated from the conditioned media of Ctrl and C202 cell cultures by ultracentrifugation (Fig. 1D, Supplemental Fig. 1A). As with the whole cell lysate, a ~ 50–75 kD HA-tagged protein consistent with CD63 was identified specifically in C202 EVs. We confirmed the presence of EVs in ultracentrifuge preparations by transmission electron microscopy and loaded a consistent number of particles from each sample based on nanoparticle tracking analysis (NTA) (Supplemental Fig. 1B). Overall, these data suggest that CD63-HA positive EVs can faithfully traffic intracellularly and be secreted extracellularly.

### Biophysical characteristics/properties of DC2 epididymal epithelial cell EVs unaffected by CD63-HA expression

Previous groups have utilized genetic strategies to label EVs endogenously (e.g., tetraspanins fused with fluorescent or bioluminescent proteins); however, the overexpression of these proteins, driven by exogenous promoters, combined with the large size of the tags raises questions about how these transgenes can impact endogenous EV production^37–45^. Overexpression of tetrapanins, and specifically CD63, can generate off-target effects at the level of EV production and even changes in physiology^37–39,42^. In fact, depending on the specific promoter used, overexpression of CD63-GFP fusion proteins in transgenic rodent models can have dramatic effects in vivo, ranging from changes in kidney function to embryonic lethality^37,38^.

**Figure 2:**
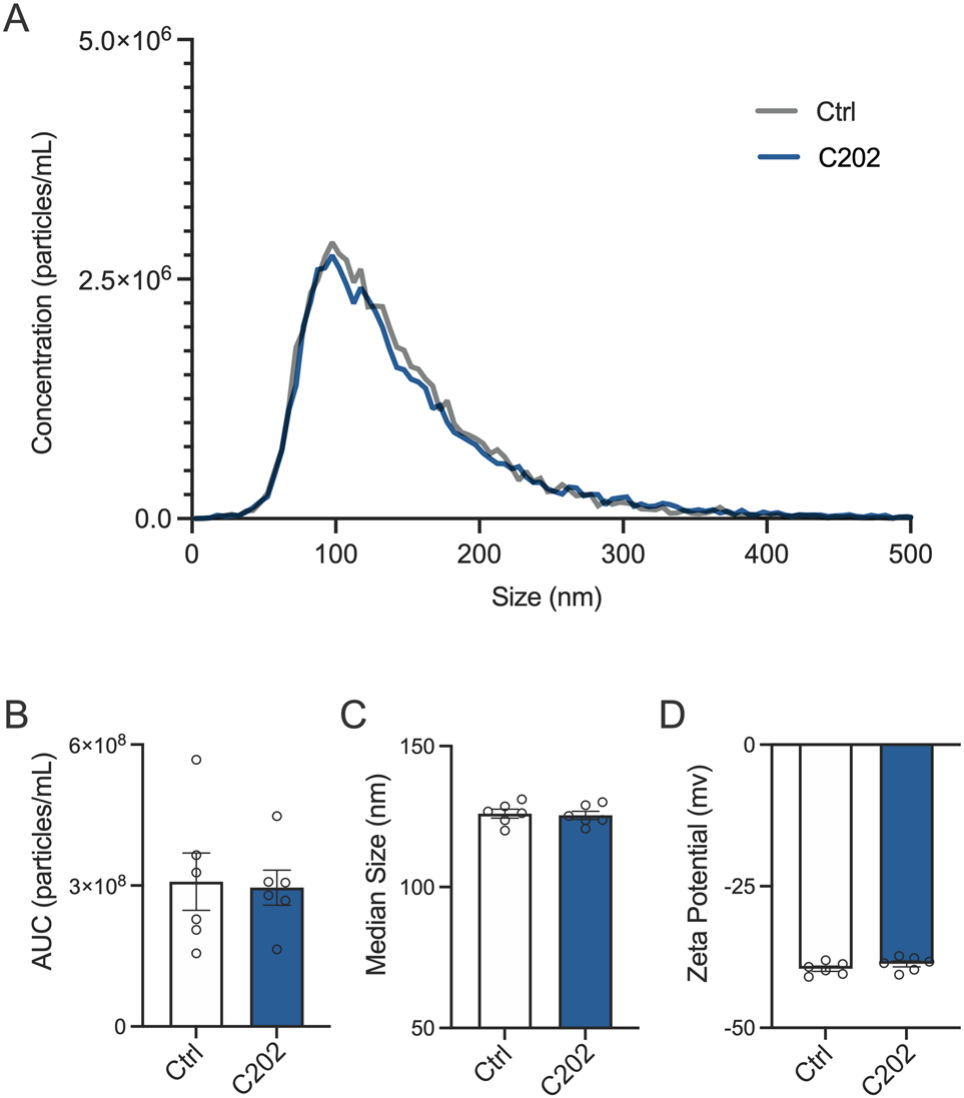
CD63-HA expression does not alter the secretion, size, or surface charge of DC2 epididymal epithelial cell extracellular vesicles (EVs). (**A**) Plots of the size distribution of EVs isolated from Ctrl and C202 DC2 conditioned media by ultracentrifugation overlap with identical single Gaussian peaks of 99.5 nm. (**B**) Area under the curve (AUC) analysis of the size distribution confirms no statistical difference in EV production between Ctrl and C202 cell lines. (**C,D**) ZetaView nanoparticle tracking also confirmed EVs secreted by Ctrl and C202 DC2 cells are indistinguishable based on median size (**C**) or surface charge (zeta potential). N = 6 samples isolated from conditioned media collected from distinct cell cultures. Data are mean ± SEM. Group means were assessed by student’s t-test, α = 0.05.

Our knock-in/homologous recombination strategy allows for the genomic context of the CD63 transgene, with its regulatory elements and regulation of CD63 transgene to remain intact. Use of the endogenous promoter should allow us to avoid dramatic changes in EV production observed with synthetic promoters used in other constructs. To determine whether the expression of CD63-HA alters overall EV production, size, or surface charge, we analyzed the size distribution, and zeta potential of EVs isolated from Ctrl and C202 DC2 epididymal epithelial cell cultures by NTA with the ZetaView instrument. Plots of the size distribution of EVs isolated from Ctrl and C202 DC2 conditioned media essentially overlap with identical single Gaussian peaks of 99.5 nm (Fig. 2A). Analysis of non-conditioned media processed for EV isolation in parallel (N = 3 samples) shows minimal EV contamination from exogenous components of the media used for the cell cultures (e.g., fetal bovine serum) (Supplemental Fig. 2)^59^. Area under the curve (AUC) analysis confirm no difference in EV production between Ctrl and C202 cell lines [t(10) = 0.18, p = 0.86] (Fig. 2B). There was also no effect of cell line on the median diameter of isolated EVs [t(10) = 0.30, p = 0.77] (Fig. 2C). EVs carry a negative surface charge due to their membrane constituents. This can be measured as zeta potential, which is a key physical property of EV stability in solution, as a loss in this negative surface charge can promote their aggregation^60^. Though the topology of CD63 ensures the carboxy terminal HA tag is oriented within the interior of labeled vesicles, the HA tag is positively charged and might adversely impact the surface charge of EVs directly or indirectly, via changes in the membrane lipid or protein content. There was no difference in the zeta potential of EVs isolated from C202 conditioned media compared to Ctrl DC2 cultures [t(10) = 1.26, p = 0.24] (Fig. 2D).

### CD63-HA expression does not affect the tetraspanin or miRNA content of DC2 epididymal epithelial cell EVs

**Figure 3:**
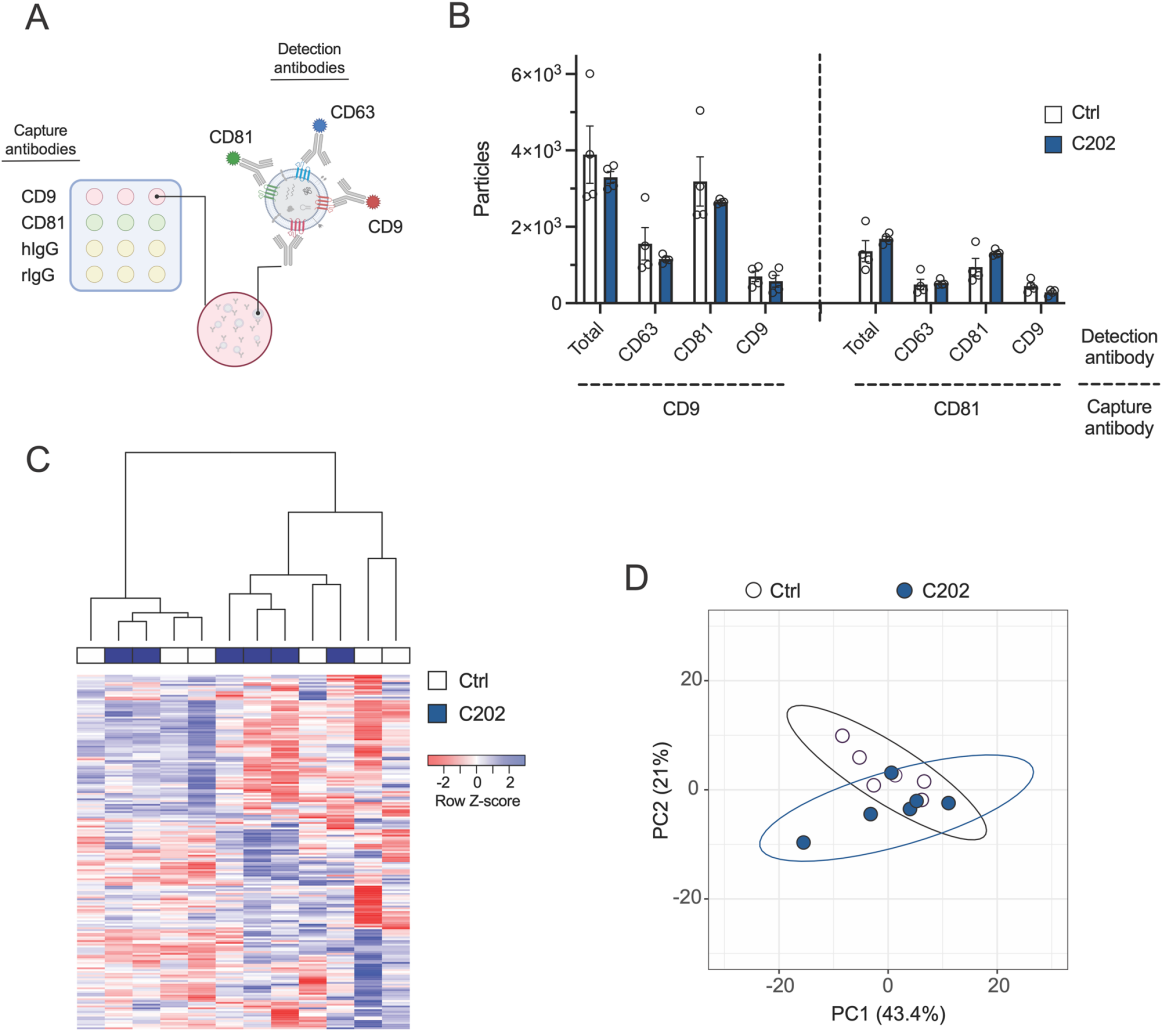
CD63-HA expression does not alter the tetraspanin composition or miRNA cargo of secreted extracellular vesicles (EVs). (**A**) Diagram of ExoView single particle interferometric reflectance imaging sensor technology (SP-IRIS) for the analysis of EV tetraspanin composition. An EV sample was applied to a silicon chip printed with an array of spots coated with capture antibodies raised against mouse tetraspanin EV protein markers (CD81 and CD9) and negative isotype controls (hamster and rat IgG). Captured EVs were then labeled with fluorescent antibodies directed against the mouse tetraspanins (CD9-AF488, CD81-AF555, and CD63-AF647). Spots with captured and labeled particles were imaged by interferometric reflectance for size and count, and fluorescent imaging to detect antibody labeling of individual EVs (**B**) ExoView SP-IRIS analysis of EVs isolated from control (Ctrl) and CD63-HA positive (C202) DC2 epididymal epithelial conditioned media identified no differences in the number of total, CD63-, CD81-, or CD9-positive EVs captured by spots printed with antibodies for CD9 or CD81. N = 4 samples isolated from conditioned media collected from distinct cell cultures. Particle counts were averaged across 3 identical spots on each chip/sample and displayed as mean ± SEM. Group means were assessed by student’s t-test, α = 0.05. (**C**) Heatmap visualization of miRNA expression of 195 total miRNA across EV samples demonstrated the lack of overall differences in the pattern of miRNA expression between Ctrl or C202 EVs. Hierarchical clustering of samples by Spearman correlation segregated these samples into 2 large non-exclusive clades. miRNA were clustered by pairwise Pearson. N = 6 samples isolated from conditioned media. (**D**) A principal component analysis (PCA) clustering of EV miRNA samples that is unable to distinguish between samples isolated from Ctrl or C202 cell lines. The majority of samples from each cell line (4 of 6) were contained within a region of overlap between 95% confidence ellipses for each group.

Study of EV composition demonstrates they carry various cargo, including proteins, lipids, and nucleic acids, and this content can vary widely between cells and conditions^1–3^. This composition directly affects the fate and function of EVs; therefore, any optimal EV marker must not produce changes in the cargo of tagged vesicles. We assessed the effect of CD63-HA on EV tetraspanin protein by single particle interferometric reflectance imaging sensor technology (SP-IRIS), and miRNA composition by bulk miRNA sequencing. CD9, CD63, and CD81 have been of particular utility for the isolation, characterization, and functional evaluation of EVs. Although overlap in these markers can exist across EV subtypes, they have been used to define unique vesicle populations, particularly in combination^39,51^. We assessed the impact of CD63-HA on the levels of this essential triad of tetraspanins as a potential measure of changes in the populations/subtypes of EVs secreted by DC2 epididymal epithelial cells. To address the overlap in tetraspanin markers across EV subtypes/populations that can be difficult to unravel in a diverse EV pool, we performed our analysis at the single-particle level using ExoView SP-IRIS. This platform can detect at least two different markers by capturing particles imaged by interferometric reflectance for sizing and counting onto a chip printed with antibodies for CD9, CD81, and isotype controls^61^ (Fig. 3A). An equal number of particles, determined by NTA, were applied to each chip (N = 4). Interferometric reflectance detected no effect of cell line on the total number of EVs captured by CD9 [t(6) = 0.78, p = 0.47] or CD81 [t(6) = 1.14, p = 0.30] spots (Fig. 3B). Fluorescent imaging detected no effect of cell line on the total number of: CD63-positive EVs captured by CD9 [t(6) = 0.93, p = 0.39] or CD81 [t(6) = 0.30, p = 0.78] spots, CD81-positive EVs captured by CD9 [t(6) = 0.83, p = 0.44] or CD81 [t(6) = 1.63, p = 0.15] spots, or CD9-positive EVs captured by CD9 [t(6) = 0.61, p = 0.56] or CD81 [t(6) = 1.86, p = 0.11] spots.

Studies from numerous cell types have shown miRNAs are commonly packaged into EVs and secreted to mediate various functions in recipient cells^1–3^. We previously demonstrated DC2 epididymal epithelial EV miRNA complement responds dynamically to prior corticosterone exposure and that sperm incubated with these EVs produce offspring with altered neurodevelopment^27^. Therefore, it is important the expression of the CD63-HA transgene does not affect the miRNA content of labeled EVs. We assessed this possibility using bulk miRNA sequencing of EVs secreted by DC2 cells (N = 6 samples per cell line). To control for potential miRNA contamination from exogenous components of the media used for the cell culture (e.g., fetal bovine serum), non-conditioned media samples were processed for EV isolation and miRNA sequencing in parallel (N = 3 samples)^59^. After filtering for features consistently present across samples (CPM ≥ 2 in at least 6 samples), we identified 195 total miRNA in DC2 EVs. Clustering of samples based on miRNA transcript expression, post-filtering and TMM normalization, identified the non-conditioned media samples as outliers, suggesting any similarity in EV miRNA content between cell lines is unlikely driven by background levels of miRNA present in exogenous EVs or miRNA-protein complexes (Supplemental Fig. 3); therefore, these negative control samples were excluded from further analysis. A heatmap of total DC2 EV miRNA expression is shown in Fig. 3C to visualize expression patterns across samples. In this heatmap, miRNA are clustered by pairwise Pearson correlation (rows) and samples are clustered using Spearman correlation (columns), which gives equal weight to highly vs lowly expressed features (i.e., miRNA). The clustering of samples segregates the samples into 2 large non-exclusive clades, suggesting the DC2 EV miRNA content is broadly similar between Ctrl and C202 cell lines. To confirm this, we then performed an unbiased multivariate principal component analysis (PCA) of these DC2 miRNA expression data. A plot of samples across the PC1 and PC2, which account for 43.4% and 21% of total variation in miRNA expression between samples respectively, is displayed in Fig. 3D. This dimensionality reduction technique fails to segregate the samples into distinct clusters. The cell line from which each EV sample was isolated and 95% confidence ellipses for each group were layered on to the plot after the unsupervised PCA analysis was completed. This confirms these DC2 EV miRNA samples fail to segregate based on cell line origin, with the majority of samples isolated from each cell line (4 of 6) contained within a region of overlap between the 95% confidence ellipses for each group.

### CD63-HA facilitates visualization of interactions between DC2 epididymal epithelial cell EVs, sperm, and IVF embryos

It is generally accepted in the field that somatic-to-germline transmission of paternal experience likely involves delivery of experience-encoding cargo to sperm that then may deliver some aspect of this cargo to the oocyte upon fertilization. Groups have visualized bulk labeled cargo (e.g., biotinylated EV proteins or metabolically labeled RNA) delivery to sperm in vitro, however, in using an exogenous labeling strategy of isolated EV preparations, questions as to the differences in the behavior of specific subsets of EVs (of different cell type or biosynthetic origin) remain^9,14–16^. In addition, the delivery of epididymal EV cargo to oocytes at fertilization to shape the embryonic epigenome and development has only been inferred from assessments of offspring outcomes following experimental manipulations of the epididymal epithelium^26,27,31^. We used fluorescent microscopy and immunogold-TEM to visualize interactions between CD63-HA-labeled EVs with sperm. We then examined the biological question as to whether sperm could transfer these EVs at fertilization and would be detectable in embryos generated with these sperm via in vitro fertilization (IVF).

**Figure 4:**
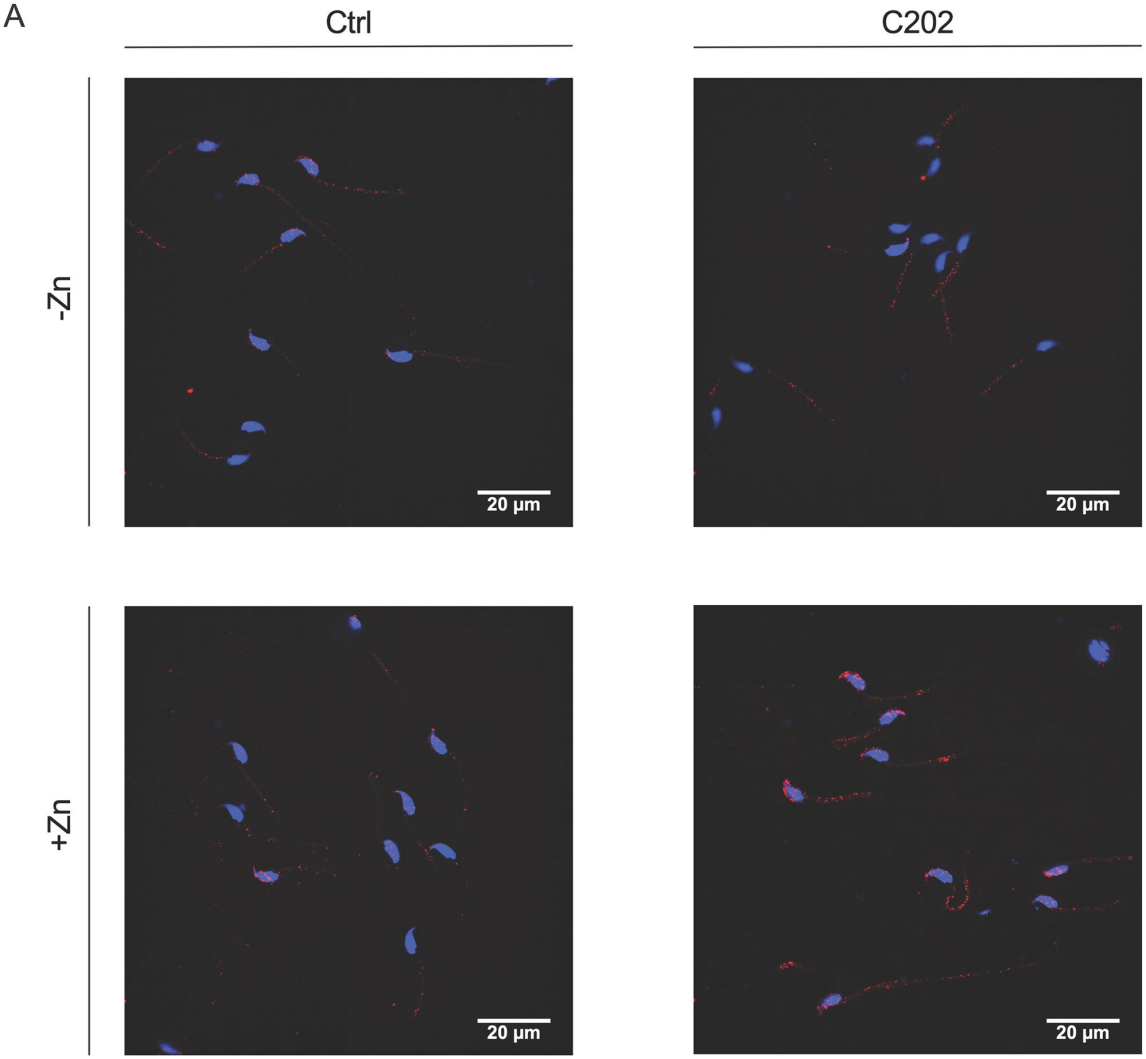

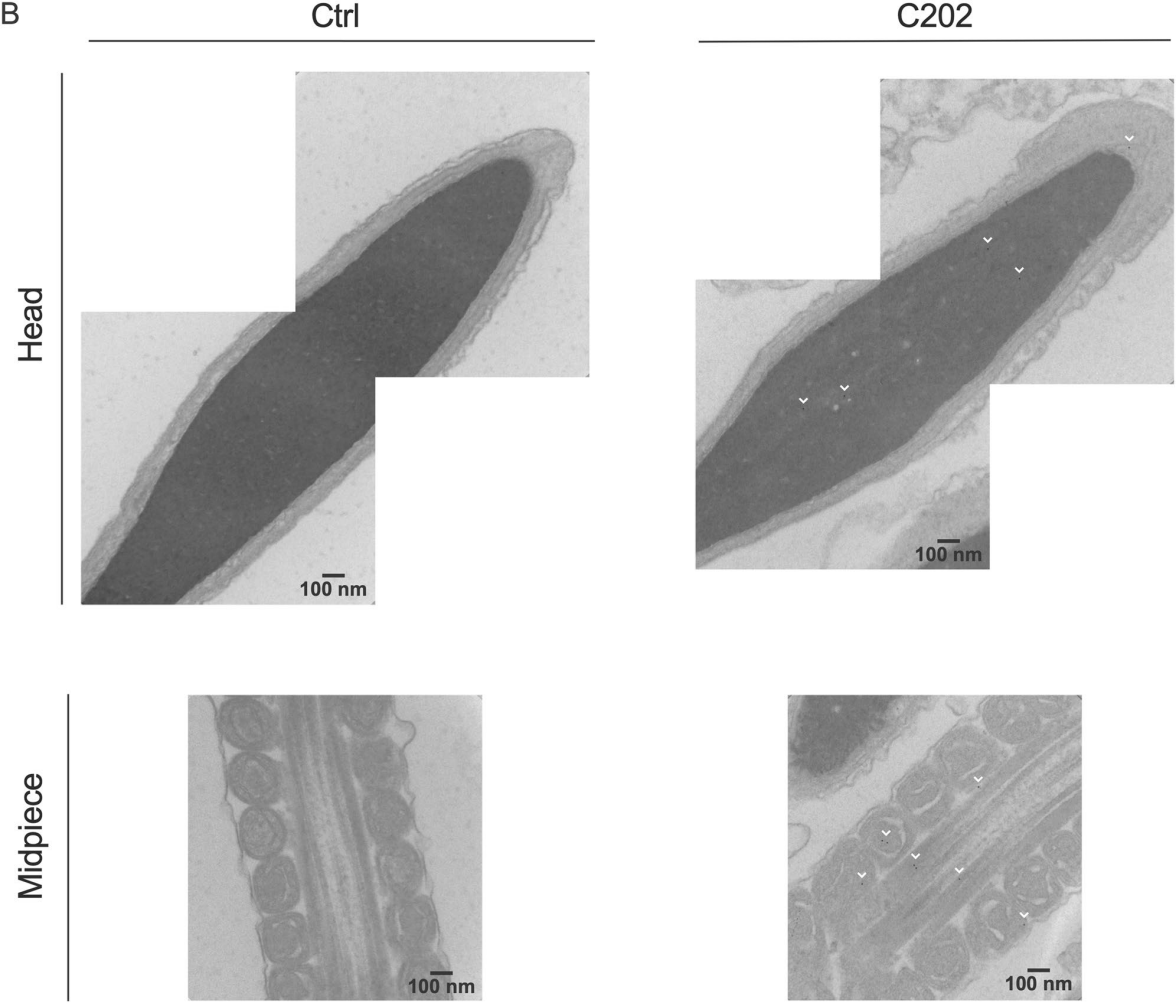
CD63-HA facilitated visualization and confirmation of interactions between DC2 epididymal epithelial cell EVs and sperm. (**A**) Immunofluorescent confocal microscopy identified anti-HA staining (red) in the head (blue) and midpiece of sperm incubated with EVs isolated from conditioned media from CD63-HA EVs (C202), but not Ctrl EVs. As expected, the presence of EVs as detected with this anti-HA staining in sperm was dependent on media supplementation with 1 mM ZnCl to mimic the intraluminal environment of the epididymis. (**B**) Immunogold transmission electron microscopy confirmed the presence of CD63-HA EVs with sperm incubated specifically with C202 EVs at the resolution of single epitopes (labeled with white arrow heads).

Confocal immunofluorescent imaging identified anti-HA staining in sperm following incubation with EVs isolated from CD63-HA positive C202 cell cultures (Fig. 4A). As expected, this staining was reduced in sperm incubated with EVs isolated from conditioned media of Ctrl cells. Consistent with foundational work on the delivery of bovine epididymal EV cargo to sperm by Sullivan et al., which has since been replicated in mouse models, anti-HA staining of sperm following CD63-HA EV incubation was concentrated in the sperm head and midpiece and was dependent on the addition of Zn^2+^ in the incubation media at a concentration reflective of the intraluminal environment of the epididymis (Fig. 4A)^9,11,14^. No-Primary antibody control samples showed the level of background staining for the anti-HA in the absence of the HA primary antibody in control and C202 sperm samples (Supplemental Fig. 4). We confirmed the presence of CD63-HA in sperm incubated with C202 EVs at the resolution of single epitopes using anti-HA immunogold transmission electron microscopy (IEM) (Fig. 4B).

**Figure 5:**
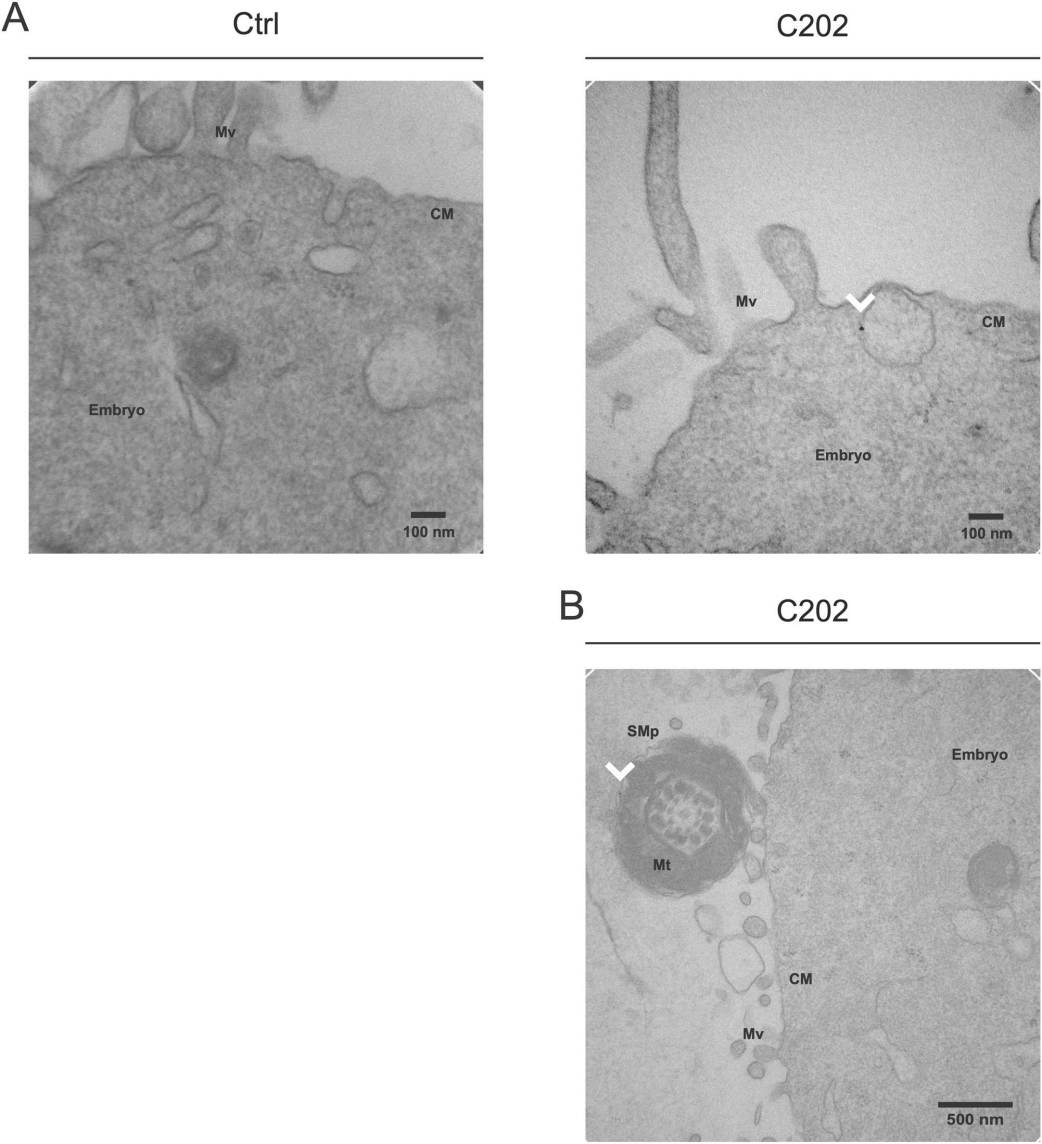
CD63-HA facilitates visualizing evidence of the transfer of DC2 epididymal epithelial cell EV cargo to fertilized embryos. (**A**) Immunogold transmission electron microscopy (IEM) confirmed anti-HA staining in 1-cell embryos generated by IVF using sperm previously incubated with CD63-HA EVs (C202), but not Control (Ctrl), at single epitope resolution (white arrow heads). (**B**) IEM also captured anti-HA staining on a sperm midpiece (SMp) found adjacent to an embryo, supporting continuous detection of EV cargo following sperm-mediated EV transfer at fertilization. CM: cell membrane, Mt: mitochondria, Mv: microvilli, SMp: sperm midpiece.

While studies have visualized the transfer of EV cargo to sperm, the subsequent delivery of this cargo at fertilization has only been inferred, often from changes in the embryo transcriptome or effects on offspring phenotypes later in development^5,13,14,26,27,31^. Therefore, we performed IVF using sperm incubated with our CD63-HA EVs and processed single-cell embryos for anti-HA IEM. Electron microscopy identified anti-HA staining, with single epitope resolution, specifically in embryos fertilized with sperm incubated with EVs isolated from CD63-HA positive C202 cells (Fig. 5A, Supplemental Fig. 5). Of the 26 embryos from C202 HA EV incubation examined, 20 showed at least one gold particle. We also captured a micrograph depicting a sperm carrying CD63-HA in proximity of an embryo following IVF (Fig. 5B). This further supports the ability for sperm to deliver EV cargo to the oocyte at fertilization and to still be detectable in the early developing embryo, at least in the context of IVF. Perhaps the relevant potential signal originating from a paternal source is not limited to a single sperm, but instead includes the entire composition of EVs associated with the thousands of sperm that are able to reach the oocyte and deliver cargo that may impact reproductive processes and ultimately the course of development.

## Conclusion

In the current studies we describe a novel CD63-HA transgene, which when combined with currently available Cre-recombinase alleles, should permit the cell type and/or temporally specific labeling of EVs without affecting their endogenous functions. We confirmed this in a stably transfected immortalized DC2 epididymal epithelial cell line, demonstrating: 1) following Cre-recombination, the CD63-HA transgene expresses an HA-tagged protein product that is packaged into secreted EVs; 2) the expression of CD63-HA has no effect on the production, size distribution, or surface charge of secreted EVs; and 3) an HA-tagged CD63 does not alter the tetraspanin (CD9/CD81/CD63) or miRNA content of EVs. Finally, we demonstrate the possible utility of HA-tagged CD63 positive EVs in visualizing interactions between epididymal epithelial cell EVs, sperm, and embryos generated by in vitro fertilization. As such, this transgene will serve as a valuable tool with applications in the study of EV function across biomedical fields.

Despite the vast and fast-growing EV biology across fields in preclinical models and clinical biomarkers, critical questions remain about the underlying biological mechanisms by which EVs function, especially in delivery of information important in shaping intergenerational outcomes. Hampered by a current lack of tools, in vivo tracking of EVs should prove particularly valuable as the field moves beyond defining basic physical and molecular parameters of these novel nanoparticles toward developing insight into their trafficking, distribution, cellular targeting, uptake, and signaling mechanisms^32,33^.

## Methods and materials

### DC2 epididymal epithelial cell culture

Immortalized mouse distal caput epididymal epithelial (DC2) cells were purchased from Applied Biological Materials and cultured as previously described^54^. Briefly, cells were seeded on cell culture plates coated in collagen type 1, rat tail (Sigma, C3867) at a concentration of 5 µg/cm^2^. Cells were grown in Iscove’s modified Dulbecco’s medium (IMDM) (Gibco, 12440-053) supplemented with 10% fetal bovine serum (Gibco, 26140079), 100 U/mL penicillin and 100 µg/mL streptomycin (Gibco, 15140122). DC2 cultures were maintained in humidified conditions at 33 °C and 5% CO_2_.

### Generating a DC2 epididymal epithelial cell line stably transfected with CD63-HA targeting construct

Preparation of a targeting construct for the mouse CD63 gene with the structure “loxP-exon8-loxP-FRT-PGK-gb2-neo-FRT-exon8/3xHA-tag” was generated by Gene Bridges (Heidelberg). This CD63-HA targeting construct was introduced into DC2 cells by electroporation-based transfection using the Amaxa Nucleofector II system (Amaxa Biosystems). Transfected cells were grown under antibiotic selection pressure with 0.35 mg/mL Geneticin sulfate (Gibco, 10131035) for 8 weeks. Polyclonal transfected cells were then plated at low density, such that following several rounds of replication, isolated clonal colonies of cells could be identified visually, overlayed with agarose, and picked for expansion (Lindberg Lab online protocol, http://thelindberglab.com/cloning-cells-with-the-agarose-method/). This process of transfection followed by selection of clonal cell lines was repeated sequentially for transient expression plasmids encoding Flipase and Cre recombinase. PCR characterization of the CD63 loci was performed with flanking primers (5′-TGGAGGTGAAGAATGCTGTG-3′ and 5′-CTGGAGAATCCACTCCATGAAA-3′). For selected cell lines, this PCR product was gel purified with MinElute Gel Extraction Kit (Qiagen, 28604) and cloned using the Zero Blunt TOPO PCR Cloning Kit, with One Shot TOP10 Chemically Competent E. coli cells (Invitrogen, K280020) according to the manufacturers’ instructions. Following expansion, the resulting plasmid was isolated using the Zyppy Plasmid Miniprep Kit (Zymo Research, ZD4019) and submitted for Sanger sequencing.

### Extracellular vesicle (EV) isolation

EVs were isolated from conditioned media using differential ultracentrifugation^64^. DC2 cells were plated in T-175 flasks in media with standard 10% FBS and allowed to grow to 80% confluency. At this point, this media was removed, the cells were rinsed in warm PBS, and 35 mL of fresh media containing 10% EV-depleted FBS (System Biosciences, EXO-FBS-250A-1) was added. Conditioned media (33 mL) was then collected every 3 days for a total of 3 collections over 9 days. Immediately following collection, non-adherent/dead cells were pelleted from the condition media by centrifugation at 2000×*g* for 10 min, then 30 mL of this cleared conditioned media was transferred to clean tubes and stored at 4 °C for no more than 48 h before proceeding to ultracentrifugation. Conditioned media was ultracentrifuged in thickwall polycarbonate tubes (Beckman Coulter, 355631) using a Beckman Coulter SW-32 Ti rotor and Optima LE-80 ultracentrifuge cooled to 4 °C. Cellular debris was removed by centrifugation at 9000 RPM (avg 10,000×*g*) for 30 min. The supernatant was transferred to clean tubes and centrifuged at 28,500 RPM (avg 100,000×*g*) for 90 min. The supernatant was then removed by aspiration and discarded, the pellet was resuspended in 25 mL of cold filtered (0.22 µm) PBS, then centrifuged for an additional 90 min at 28,500 RPM. Following this final spin, the supernatant was removed, the pellet was resuspended in 250 µL of cold filtered PBS, then stored at − 80 °C. For each collection, aliquots of fresh non-conditioned media were processed in the same way to serve as controls for potential EV contamination from components of the culture media (e.g., EV-depleted FBS). After EVs were isolated from all collections, samples from the three timepoints for each culture flask were pooled and these EV pools were aliquoted in appropriate volumes for downstream analyses before final storage at − 80 °C, to minimize freeze–thaw cycles. The experimental unit is the pooled EV sample generated by combining 3 EV samples isolated from conditioned media collected at 3 timepoints (every 3 days) from a single cell culture flask. Therefore, the experimental unit is the flask of cultured cells.

### Protein extraction and western immunoblotting

Protein was extracted from cell pellets or isolated EVs in radioimmunoprecipitation assay (RIPA) buffer (EMD Milllipore, 20-188) with 0.1% SDS and cOmplete EDTA-free protease inhibitor (Roche Diagnostics, 11836170001). Samples were shaken for 15 min at 3000 RPM at 4 °C using a Disruptor Genie (Scientific Industries) and stored at − 80 °C. For cellular extracts, samples were centrifuged at 12,000×*g* for 10 min at 4 °C and protein was quantified using BCA assay (Thermo Scientific, 23227). For immunoblots, gel electrophoresis was performed using NuPAGE 4–12% Bis–Tris gel (Invitrogen, 0321) loaded with a standard quantity of protein for cell extract samples or protein extracted from a fixed number of particles (2.1 × 10^10^ assessed by NTA) for EV samples. After transfer of proteins to a nitrocellulose membrane (Life Technologies, LC2000), membranes were blocked with Intercept blocking buffer-TBS (LI-COR, 927-60001) with 0.1% Tween-20. Whole cell protein extracts were probed with rabbit anti-HA mAB (C29F4) (1:500; Cell Signaling Technology, 3724), and mouse anti-beta Actin (1:1000; abcam, ab8226), followed by incubation in IRDye 800-conjugated donkey anti-rabbit secondary (1:2000; LI-COR, 926-32213) and IRDye 680RD-conjugated goat anti-mouse secondary (1:2000; LI-COR, 925-68070). Isolated EV protein extracts were probed with rabbit anti-HA mAB (C29F4) (1:500; Cell Signaling Technology, 3724), followed by incubation in IRDye 800-conjugated donkey anti-rabbit secondary (1:2000; LI-COR, 926-32213). We and others have previously utilized and shown specificity of this anti-HA antibody (73–75).

### Nanoparticle tracking analysis (NTA)

The concentration, size, and surface charge (zeta potential) of isolated EVs were measured using a ZetaView BASIC Nanoparticle Tracking Analysis Microscope (Particle Metrix). EVs were diluted in freshly filtered (0.22 µm) water to achieve ~ 200 particles per frame. The size and concentration of particles were measured by scanning 11 cell positions, with 30 frames per position, over 2 cycles. Surface charge was measured across 11 positions. Sensitivity for video acquisition was set to 80, and shutter speed was set to 100. Native ZetaView software (version 8.05.14) was used to analyze all videos, with minimum brightness 20, minimum area 10, and maximum area 1000. Cell line comparisons of particle concentration (AUC), size, and zeta potential were analyzed by unpaired t tests. Statistical analyses and visualizations were performed using Graphpad Prism (version 9.4.0).

### Single particle interferometric reflectance imaging (SP-IRIS)

The tetraspanin composition of isolated EV samples was assessed using NanoView’s SP-IRIS technology with their ExoView Mouse Tetraspanin kit according to the manufacturer’s instructions (NanoView Biosciences, EV-TETRA-M2). Samples were diluted in a proprietary buffer such that, for each sample, 5 × 10^8^ particles in a volume of 35 μL was applied to silicon chips coated with individual antibody spots against mouse CD9 and CD81 as well as negative isotype. After overnight incubation, chips were washed, then incubated for one hour at room temperature with a cocktail of fluorescent antibodies (anti-CD9-CF488, anti-CD63-CF647, and anti-CD81-CF555). Following a final set of washes, chips were imaged with the ExoView R100 scanner by interferometric reflectance imaging and fluorescent detection (NanoView Biosciences). Data processing was performed with the manufacturer’s ExoView Analyzer 3.0 software. Cell line comparisons of particle counts were analyzed by multiple unpaired t tests. Statistical analyses and visualizations performed using Graphpad Prism (version 9.4.0).

### EV miRNA sequencing

RNA, enriched for small RNA, was isolated using Zymo Research’s Direct-zol RNA MicroPrep kit (Zymo Research, ZR2060) according to manufacturer’s instructions. RNA concentration and quality were assessed using Agilent’s small RNA chips (Agilent Technologies, 5067-1548) run on a Bioanalyzer 2100 (Agilent Technologies). The small RNA content of sperm samples was analyzed by small RNA sequencing. Libraries were constructed using the TruSeq small RNA Library Prep Kit (Illumina, RS-200-0012/RS-200-0024) with 25–50 ng of small RNA according to the manufacturer’s protocol. Post-PCR cleanup and size selection for products > 100 bp was performed using Agencourt AMPure XP bead purification (Beckman Coulter, A63880) using a bead to sample ratio of 1.8:1 (v:v). Library size distribution and quantification was performed on a TapeStation 4200 (Agilent Technologies) using their High Sensitivity D1000 screentape (Agilent Technologies, 5067-5585). Individually barcoded libraries were pooled to achieve ~ 10 million reads per sample and sequenced on an Illumina NextSeq 550 (75-bp single-end) using the NextSeq 500/550 High Output Kit v2.5 flow cell (Illumina, 20024906).

Our small non-coding RNA sequencing (sncRNASeq) analytical pipeline was designed using the snakemake framework and is available via GitHub at https://github.com/acshetty/sncRNA-seq-analysis^65^. The miRBase 21 reference transcriptome sequences were downloaded in FastA format [Kozomara.2014]. Using ‘index_ref’ component, the reference FastA file was indexed using the ‘bowtie-build’ from the Bowtie short read aligner software^66^. Our sequencing reads were longer than the average size of most miRNA, which may result in the inclusion of adapter sequence at the 3′-end of the read sequence; therefore, the ‘trim_fq’ component was invoked to remove trailing adapter sequence using the Trimmomatic tool^67^. After trimming, reads shorter than 15 nucleotides were discarded before downstream analyses. The trimmed reads for each sample were then aligned, using the ‘align_reads’ component, to the reference transcriptomes using the Bowtie short read aligner^66^. Reads were aligned allowing for 2 mismatches and a seed length of 15 nucleotides. Raw expression values were computed using the ‘compute_expr’ component based on the number of reads aligned to the reference file. Subsequent data processing, statistical modeling, and visualization were performed in the R software environment (version 4.1.3)^68^. Expression of miRNA were adjusted for differences in library sequencing depth to generate counts per million reads (CPM) following TMM normalization using the Bioconductor package ‘edgeR’ (version 3.36.0)^69^. Features were retained for analysis if they were expressed at ≥ 2 CPM in at least 6 samples. Multivariate analyses were performed with the base R package ‘stats’^68^. Data visualizations were generated using ‘ggplot2’ (version 3.3.6)^70^.

### Animals

All mice were in-house mixed C57BL/6:129 mice aged 8–12 weeks. Animals were housed in a 12:12 light:dark cycle at ambient temperature with ad libitum access to food, water and environmental enrichment (nesting material). All procedures were approved by the University of Maryland Baltimore Institutional Animal Care and Use Committee. All methods were carried out in accordance with relevant guidelines and regulations and reported in accordance with the ARRIVE guidelines.

### Cauda sperm collection

Males were rapidly decapitated under isoflurane anesthesia. Immediately following euthanasia, an incision was made in the abdomen of 8–12 week old experienced Bl6 × 129 male mice and testes were exposed by pulling the testicular fat pad. Cauda epididymides were dissected and transferred into a 500 µL droplet of Embryomax M2 medium (Sigma, MR-015) pre-gassed in a 5% CO_2_ incubator and maintained at 37 °C under mineral oil. Using small scissors, 5–7 incisions were made through the cauda to permit sperm cells to escape into the solution. Following a 30 min incubation, excess tissue was removed, and sperm were collected into a 1.5 mL tube using a 200 µL wide orifice pipet tip (USA Scientific, 1011-8410). Separate aliquots from each sperm sample were diluted and fixed in 10% neutral buffered formalin (Sigma, HT501128) for counting in a hemacytometer.

### Incubation of cauda epididymal sperm with DC2 EVs

Co-incubation of epididymal sperm and isolated EVs was performed using methodology originally optimized for the in vitro transfer proteins between bovine epididymal EVs and sperm, and later adapted for mouse models^9,11^. Cauda sperm (2 × 10^6^) were incubated with EVs isolated from DC2 epididymal epithelial cell conditioned media (4 × 10^9^ total particles) in modified M2 buffer (pH 6.5), with or without supplemental ZnCl_2_ (1 mM), for 2 h at 37 °C in 5% CO_2_. Following incubation, sperm were used immediately for IVF or immunocytochemistry.

### Immunocytochemistry and confocal microscopy

DC2 cells were plated in a 24-well black frame plate with glass-like polymer bottom (Cellvis, P24-1.5P) and cultured as described above to 80% confluency, then fixed with 4% formaldehyde (Thermo Scientific, 28908) in PBS for 20 min at 4 °C. Fixed cells were incubated in blocking buffer [5% BSA (Sigma, A3059), 0.3% Triton X-100, and 0.05% sodium azide in PBS) for 1 h at RT, then probed with rabbit anti-HA mAB (C29F4) (1:800; Cell Signaling Technology, 3724), followed by incubation with Alexa Fluor 488 labeled goat anti-rabbit IgG (1:1000; Invitrogen, A11008) for 1 h at RT. Nuclei were counterstained with Hoechst 33342 (1:2000; Invitrogen, H3570).

Following incubation with EVs, caudal sperm were placed in a μ-Slide 8 well high IbiTreat polymer coverslip plate (ibidi, 80806), allowed to dry at 37 °C for approximately 30 min, then fixed with 4% formaldehyde (Thermo Scientific, 28908) in PBS for 30 min at 4 °C. Fixed cells were incubated in blocking buffer [5% Normal goat serum (Jackson ImmunoResearch, 005-000-121) and 0.1% Saponin (Sigma, S7900) in PBS] for 1 h at RT, then probed with rabbit anti-HA mAB (C29F4) (1:250; Cell Signaling Technology, 3724) in antibody dilution buffer [1% BSA (Sigma, A3059), 0.1% Saponin (Sigma, S7900) in PBS] overnight at 4 °C, followed by incubation with Alexa Fluor 594 labeled goat anti-rabbit IgG (1:1000; Invitrogen, A11008) in antibody dilution buffer [1% BSA (Sigma, A3059), 0.1% Saponin (Sigma, S7900) in PBS] for 90 min at RT. Nuclei were counterstained with Hoechst 33342 (1:10,000; Invitrogen, H3570). No-Primary control samples were incubated as above, but without primary rabbit anti-HA mAB antibody.

Following immunostaining, DC2 cells and sperm were imaged on a Nikon W1 spinning disk microscope equipped with a 60× , 1.49 N.A. objective. Pixel acquisition was at 0.06 µm/pixel and images were digitized at 16 bits. Image display and analysis was performed using ImageJ (version 2.3.0)^71^.

### In vitro fertilization

Donor 8- to 12-week-old Bl6 × 129 females were superovulated using 7.5 IU of pregnant mare serum gonadotropin (PMSG) (BioVendor R&D, RP1782725000) at 18:00 h followed 48 h later by 7.5 IU of human chorionic gonadotropin (hCG) (Sigma, CG10). All hormone aliquots were stored at − 80 °C and thawed immediately prior to intraperitoneal injections. Females were euthanized using cervical dislocation by specifically trained personnel. Following euthanasia, cumulus-oocyte complexes (COCs) were dissected from the ampulla of the oviduct 13–16 h post hCG. Following co-incubation with EVs, cauda epididymal sperm (2 × 10^5^) were capacitated in CARD modified Human Tubal Fluid (mHTF) fertilization media (Cosmo Bio, KYD-008-02-EX) for 1 h in a 37 °C, 5% CO_2_ incubator, before the addition of freshly dissected COCs. Fertilization was permitted for 3 h, then oocytes/embryos were washed and transferred to fresh mHTF. Six hours after fertilization embryos were identified by the presence of two pronuclei and processed for immunogold transmission electron microscopy.

### Immunogold transmission electron microscopy (IEM)

As visualization of the HA tag in sperm samples using immunofluorescence has limitations related to the microscope point spread function and diffraction limits with amplification of signal from a fluorescent antibody, we also examined HA specific signal using immunogold transmission electron microscopy. Sperm and embryo samples were fixed overnight at 4 °C in 0.1 M Sorensons sodium phosphate buffer, pH 7.2 (Electron Microscopy Sciences, 11600-05) supplemented with 4% paraformaldehyde, 0.1% glutaraldehyde, and 3 mM MgCl_2_. Samples were postfixed in 1% osmium tetroxide, 1.5% potassium ferrocyanide in 0.1 M sodium phosphate for one hour on ice in the dark. Following fixation, samples were rinsed in water, dehydrated in a graded series of ethanol, and embedded in Poly/Bed 812 resin (Polysciences, 08792-1). Samples were polymerized at 60 °C overnight.

Thin sections, 60 to 90 nm, were cut with a diamond knife on a Leica UCT ultramicrotome and picked up with Formvar coated 2 × 1 nickel slot grids (Electron Microscopy Sciences, FF2010-Ni). For antibody labeling, grids were wetted with water and floated on all subsequent steps. Sections were first etched with 3% sodium meta periodate, rinsed in water before 10 mM NH_4_Cl in TBS, blocked for 30 min at room temperature [1% BSA (Sigma, A3059) and 1% Normal goat serum (Jackson ImmunoResearch, 005-000-121) in TBS], then incubated overnight at 4 °C with rabbit anti-HA mAB (C29F4) (1:50; Cell Signaling Technology, 3724). After incubation with the primary antibody, grids were brought to room temperature for 1 h, rinsed with blocking buffer and TBS, then incubated with 6 nm colloidal gold conjugated AffiniPure goat anti-rabbit IgG (1:40; Jackson ImmunoResearch, 111-195-144) at room temperature for 2 h. Grids then underwent a primary fixation (2.5% Glutaraldehyde in 0.1 M sodium cacodylate buffer) and were stained with 2% uranyl acetate. Grids were stored in a cool, dark place until imaged with a Hitachi 7600 TEM at 80 kV. Images were captured with an AMT CCD XR80 (8 megapixel camera—side mount AMT XR80—high-resolution high-speed camera) at either 50,000× or 120,000×.

### Data sharing

All raw sequencing data that supports the findings of this study will be deposited in an online data repository (e.g., NCBI’s Gene Expression Omnibus). The sncRNA alignment and quantification pipeline is available via GitHub (https://github.com/acshetty/sncRNA-seq-analysis). The data that support the findings of this study are available from the corresponding author upon reasonable request.

## Supplementary Information

Supplementary Legends.

Supplementary Figures.
