# Supplementary material for: HA-tag CD63 is a novel conditional transgenic approach to track extracellular vesicle interactions with sperm and their transfer at conception
Source: Sci Rep. 2023 Jan 13;13:707. doi: 10.1038/s41598-023-27898-5 (PMC9839718; doi:10.1038/s41598-023-27898-5)
Supplement: Supplementary file 1 — Supplementary Legends. [file 41598_2023_27898_MOESM1_ESM.docx]

**Supplemental Figure 1. Full length western blot confirming HA labeling of CD63-HA stably transfected DC2 epididymal epithelial cells and the extracellular vesicles (EVs) secreted following Cre-recombinase expression. (A)** Full length western blot of whole cell lysates (cells) detects a ~50-75 kD HA-tagged protein (green) specifically in DC2 cells stably transfected with CD63-HA (C202) and not in non-transfected DC2 control cells (Ctrl). The size range is consistent with the variably glycosylated CD63 protein. Actb protein (red) was used as a loading control. Western blot detects an HA-tagged protein (green) of ~50-75 kD consistent with CD63 in EVs isolated from conditioned media from C202, but not Ctrl, demonstrating CD63-HA is successfully packaged into EVs for secretion. Total protein was extracted from a constant number of EVs as determined by nanoparticle tracking. **(B)** Negative stain transmission electron microscopy confirmed the presence of EVs with expected size and heterogeneity in samples isolated from DC2 conditioned media by differential ultracentrifugation.

**Supplemental Figure 2. Minimal contamination of EV samples with particles from exogenous sources identified in non-conditioned media samples by nanoparticle tracking analysis (NTA).** Visualizing the size (diameter) distribution of EVs isolated from negative control non-conditioned media (nCM) compared to media conditioned by control (Ctrl) and CD63-HA transfected (C202) DC2 epididymal epithelial cells confirms there is minimal EV contamination from exogenous components of the media (e.g., EV-depleted fetal bovine serum).

**Supplemental Figure 3. Minimal contamination of EV samples with miRNA from exogenous sources identified in non-conditioned media samples by miRNA sequencing.** Principal component analysis clustering of EV samples based on the expression of 195 total miRNA identified samples isolated from negative control non-conditioned media (nCM) as obvious outliers compared to samples isolated from media conditioned by control (Ctrl) and CD63-HA transfected (C202) DC2 epididymal epithelial cells. This suggests any differences between the miRNA content of EVs secreted by Ctrl and C202 cells are unlikely to be obscured by background signal from miRNA contamination from exogenous components of the media (e.g., EV-depleted fetal bovine serum) shared across cell cultures.

**Supplemental Figure 4.** No-primary control samples for immunofluorescent confocal microscopy for anti-HA staining (red) in the head and midpiece of sperm (blue) incubated with EVs isolated from conditioned media from CD63-HA EVs (C202) or Ctrl EVs.

**Supplemental Figure 5.** **CD63-HA visualization in an additional fertilized embryo.** Similar to Figure 5, immunogold transmission electron microscopy (IEM) confirmed anti-HA staining in an additional 1-cell embryo generated by IVF using sperm previously incubated with CD63-HA EVs (C202) at single epitope resolution (white arrow heads).
