# Supplementary figures and images for: HA-tag CD63 is a novel conditional transgenic approach to track extracellular vesicle interactions with sperm and their transfer at conception
Source: Sci Rep. 2023 Jan 13;13:707. doi: 10.1038/s41598-023-27898-5 (PMC9839718; doi:10.1038/s41598-023-27898-5)

A

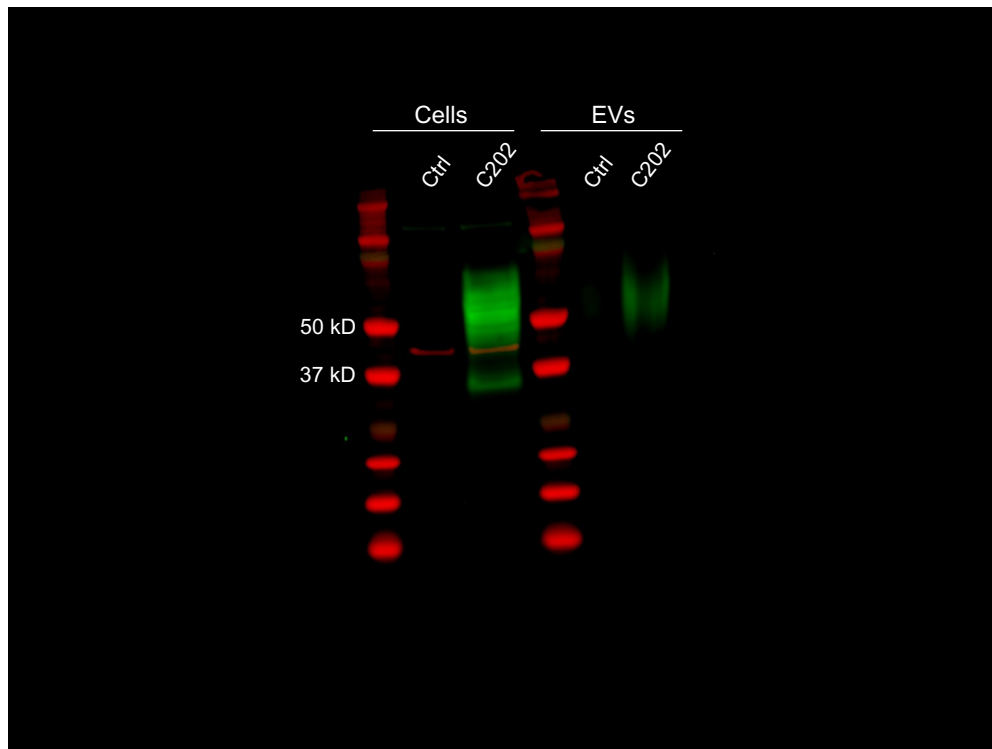

B

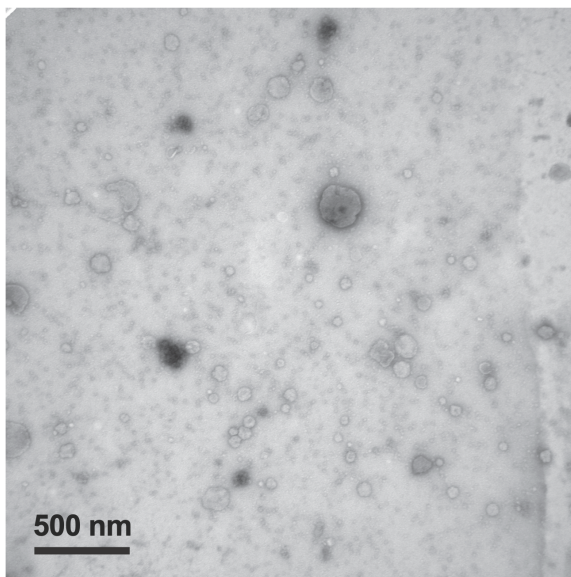

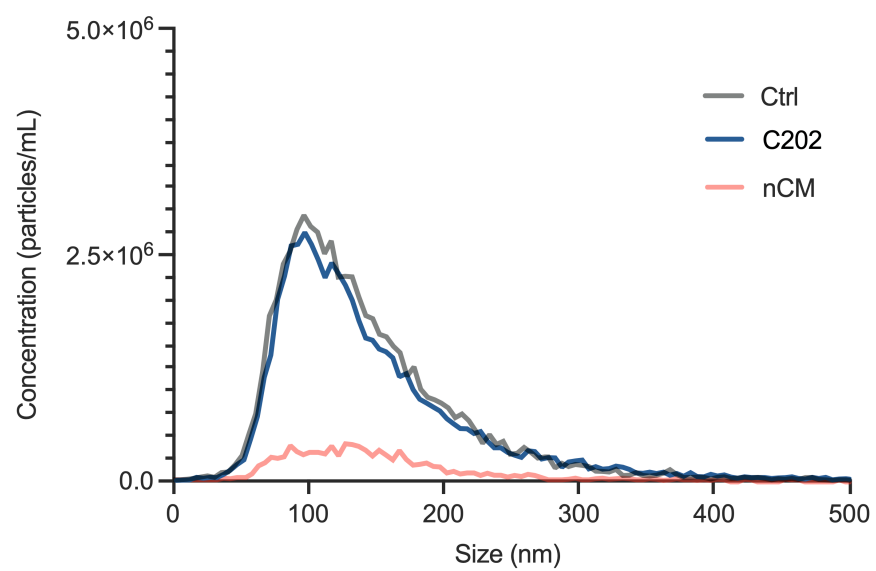

Supplemental figure 2

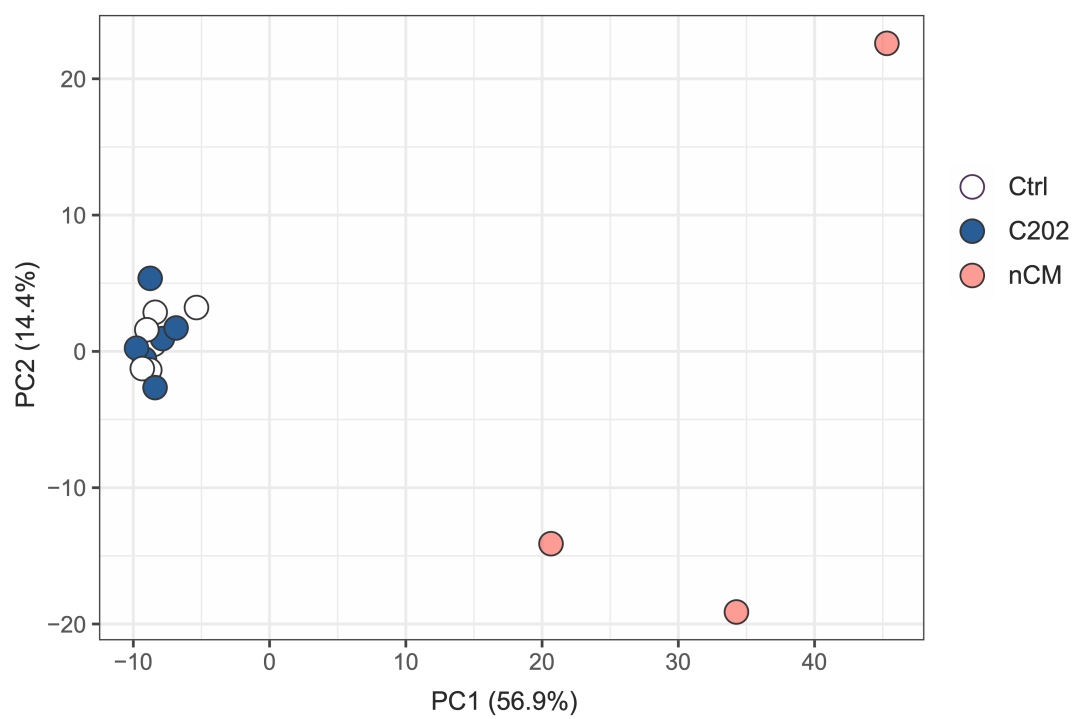

Supplemental figure 3

Ctrl

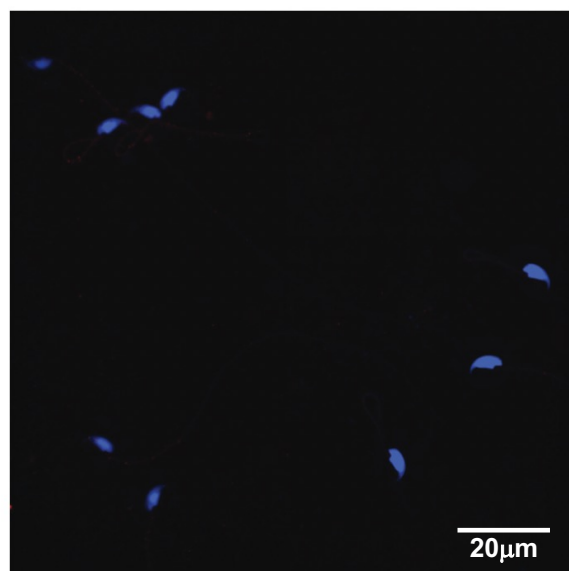

C202

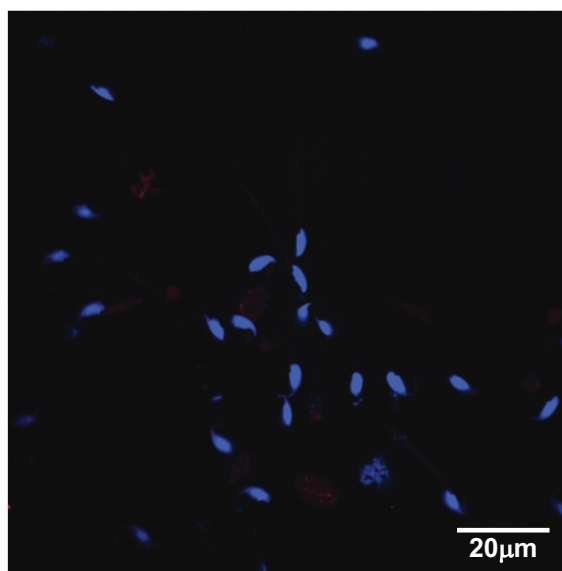

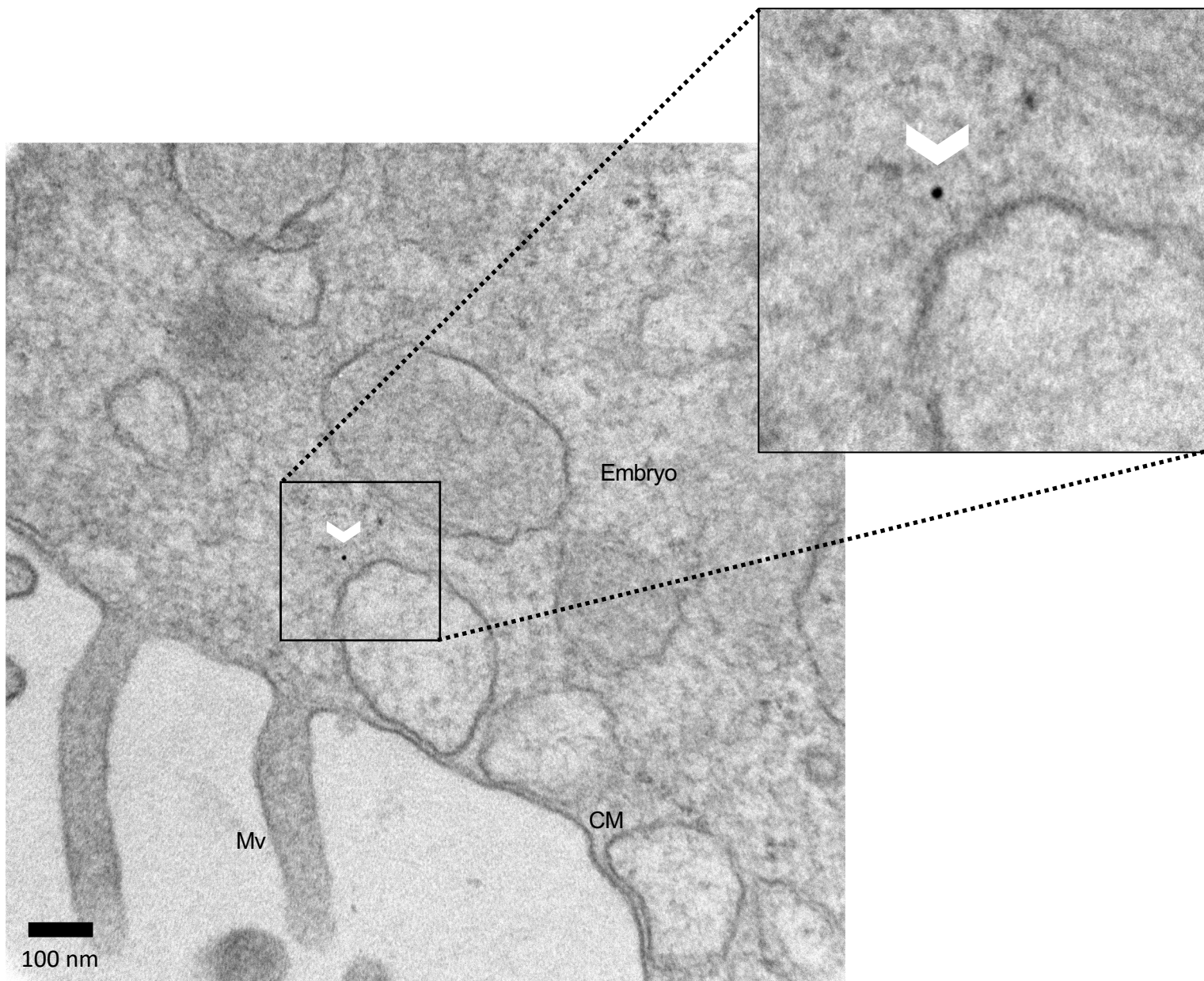

Supplemental figure 5

Supplement: Supplementary file 2 — Supplementary Figures. [file 41598_2023_27898_MOESM2_ESM.pdf]
